# Do Higher-Quality Regulatory Measures Promote a Healthier School Food Environment?

**DOI:** 10.3390/ijerph23020244

**Published:** 2026-02-14

**Authors:** Ana Carolyne Lima Lino Sandes, Ariene Silvado Carmo, Larissa L. Mendes, Mariana C. de Menezes

**Affiliations:** 1 Graduate Program in Nutrition and Health, Department of Nutrition, Federal University of Minas Gerais, Belo Horizonte 30130-100, MG, Brazil; 2 Department of Clinical and Social Nutrition, Federal University of Ouro Preto, Ouro Preto 35400-000, MG, Brazil; arienecarmo@gmail.com; 3 Department of Nutrition, School of Nursing, Federal University of Minas Gerais, Belo Horizonte 30130-100, MG, Brazil; larissa.mendesloures@gmail.com (L.L.M.); marysnut@gmail.com (M.C.d.M.)

**Keywords:** food commercialization, school canteens, school feeding, school food environment, government regulation, nutrition policy

## Abstract

**Highlights:**

**Public health relevance—How does this work relate to a public health issue?**

- This study evaluates how the presence and quality of municipal and state regulations influence the school food environment, a central element in shaping the eating habits of children and adolescents.
- It shows that school canteens located in areas without regulation present greater availability and promotion of ultra-processed foods, increasing risks related to childhood obesity and non-communicable chronic diseases.

**Public health significance—Why is this work of significance to public health?**

- This study demonstrates, based on data from 2241 canteens across 27 Brazilian capitals, that higher-quality regulations are associated with greater implementation of food and nutrition education actions, lower sales of ultra-processed foods, and reduced use of advertising.
- It provides robust evidence reinforcing the essential role of well-structured public policies in promoting healthy eating environments within schools.

**Public health implications—What are the key implications or messages for practitioners, policy makers and/or researchers in public health?**

- It recommends strengthening and expanding school regulations aligned with Federal Decree No. 11,821/2023, including active monitoring and mechanisms for social oversight.
- It highlights that the development of high-quality regulations can transform the school food environment, guiding managers and policymakers in building more effective and equitable strategies.

**Abstract:**

This present study analyzed the association between the presence and quality of regulatory measures and the promotion of healthy eating in canteens of 2241 private elementary and secondary schools located in 27 Brazilian state capitals. Three strategic axes were evaluated: food and nutrition education (implementation of actions promoting healthy eating), food commercialization (healthiness index, number of unprocessed, minimally processed or processed foods and culinary preparations based on these foods—UMPCP; ultra-processed foods and culinary preparations based on these foods—UpCP; comparison of UMPCP versus UpCP variety; and prohibition of food sales), and marketing communication strategies (advertising strategies for UMPCP and UpCP). The presence and quality of municipal and state regulations in force up to the month prior to data collection were assessed using a score, with a score ≥8 indicating higher quality. Analyses were conducted using binary logistic regression and adjusted generalized linear models in Stata 17.0. More than half of the canteens (51.1%) were located in areas without regulations, and only 17.8% had high-quality regulations. Canteens in areas with regulations, especially those with a score ≥8, had 1.73 times higher odds of implementing food and nutrition education actions, 2.49 times higher odds of prohibiting the sale of certain foods, and 36% lower odds of selling a higher variety of UpCP compared to UMPCP. The healthiness index was higher, the number of UpCP sold was lower, and the number of UMPCP sold was higher, while the adoption of advertising strategies was less frequent in canteens with higher-quality regulations. These findings indicate that the presence and particularly the quality of regulatory measures is associated with healthier school food environments, highlighting the positive impact of well-structured public policies.

## 1. Introduction

The school food environment encompasses both the spaces and infrastructure, as well as the internal and surrounding conditions of schools, where foods are made available, purchased, and consumed [1]. School canteens, in turn, constitute specific environments for the commercialization of foods and beverages for the school community. This environment directly influences the formation of dietary habits and choices among children and adolescents, considering that they spend at least one-third of their day at school, which is considered a strategic setting for health promotion interventions [2,3,4].

In Brazil, canteens are present in almost all private schools and in about one-third of public schools [5]. Studies indicate that private schools have higher sales and marketing of ultra-processed foods, which are associated with increased consumption of these products among students [6,7,8]. Consequently, regulation of the school food environment has been widely recommended by international organizations, such as the World Health Organization (WHO) [9], the United Nations Children’s Fund (UNICEF) [10], and the Organization for Economic Cooperation and Development (OECD) [11], as an essential strategy to prevent and address childhood obesity and other non-communicable chronic diseases. These institutions emphasize the importance of measures that limit the promotion and availability of ultra-processed foods while encouraging educational actions in schools.

International examples demonstrate the positive impact of such policies. In March 2025, Mexico implemented a national regulation banning the sale of ultra-processed foods in schools to combat childhood obesity and diabetes, widely recognized as a model of effective school-based intervention [12]. In the United States and Australia, research shows that policies restricting unhealthy food offerings promote greater availability of healthy options [13,14,15,16,17].

In Brazil, several cities and states have enacted legislation over the past two decades regulating food sales in schools [18,19]. A significant advancement was the publication of Decree No. 11,821/2023, which establishes national guidelines for promoting adequate and healthy nutrition in school settings. This decree, aligned with the Brazilian Dietary Guidelines, instructs states and municipalities to develop local regulations covering three strategic axes: Nutrition Education (EAN), Food and Beverage Donation and Commercialization, and Marketing Communication of Foods and Beverages [20].

Despite these advances, gaps remain regarding the implementation, monitoring, and evaluation of the effectiveness of existing regulations. The literature highlights a scarcity of studies examining whether current regulations indeed promote healthier school environments [6,19]. Previous studies have assessed the effect of regulations on food availability, but few have simultaneously addressed marketing and food and nutrition education axes [8,19]. Only one prior investigation analyzed all 27 Brazilian capitals, although it focused solely on the presence of soft drinks in canteens [21].

Given this context, there is a need to generate robust evidence on the influence of regulatory quality on the school food environment. This study aims to fill this gap by analyzing the association between the presence and quality of regulatory measures and the promotion of adequate and healthy nutrition in private school canteens across the 27 Brazilian capitals.

## 2. Materials and Methods

### 2.1. Study Design and Population

This is an ecological study with a multilevel analysis, using private elementary and secondary school canteens located in the 27 Brazilian state capitals as the unit of analysis. The study is part of the “Food Sale in Brazilian Schools” (Caeb) project https://estudocaeb.nutricao.ufrj.br (accessed on 8 January 2026), conducted between 2022 and 2024, which aimed to evaluate the sale of foods and beverages in private schools and to support policies promoting healthier dietary choices.

Data were collected on the availability, nutritional quality, prices, and marketing communication strategies of foods sold in school canteens and by street vendors around the schools [22].

This study used data exclusively from school cafeterias. The sample was defined based on the National Institute of Educational Studies and Research Anísio Teixeira (INEP) data from 2021 School Catalog [23], including all private schools offering elementary and secondary education in the capitals. Simple random sampling with an inverse sampling method was employed. When more than one canteen existed per school, all were included. Schools exclusively offering early childhood education and those with fewer than 50 enrolled students were excluded due to specific characteristics in food provision. The estimated sample size was 2077 canteens, resulting in 2241 units evaluated after exclusions. Additional methodological details are described by Canuto et al. (2025) [22].

### 2.2. Data Collection

Data were collected between June 2022 and June 2024 by trained teams under the supervision of the coordinating committee, on school days during morning and afternoon shifts, with interviews averaging 30 min. All researchers and interviewers were trained in the data collection process using an instructional manual to guide the process and a video explaining the entire content of the manual [22].

The “Instrument for the Assessment of Food Commercialization in School Canteens” https://estudocaeb.nutricao.ufrj.br/documentos/Instrumento_Cantinas.pdf (accessed on 8 January 2026 was used, whose validity and reproducibility had been previously evaluated [24] (Borges et al., 2025). Information was collected on: canteen administration type, number of customers served, number of staff, presence of a nutritionist, implementation of healthy eating promotion actions, prohibition of certain foods, and advertising strategies. An on-site audit was also conducted to assess the variety and availability of 50 food and beverage subgroups classified according to the NOVA system [25]: UMPCP: unprocessed, minimally processed, or processed foods and culinary preparations based on these foods (21 subgroups); UpCP: ultra-processed foods and culinary preparations based on these foods (29 subgroups) (Supplementary Material Table S1).

A systematic search was conducted for existing municipal and state regulatory measures governing the sale and advertising of school foods. This search was performed by two researchers via consultation of official government websites up to the month prior to the start of data collection in each city. A database was created with extracted information, including region, locality, year of publication, type of legal instrument (resolution, normative instruction, ordinance, law, or decree), whether private schools were covered, whether the regulation included school surroundings, and current status (in force or repealed). Repealed legal instruments were not analyzed in this study.

To evaluate the quality of regulatory measures, a scoring system was applied based on criteria proposed by Rocha et al. (2023) [19]. This scoring assesses whether regulations effectively promote adequate and healthy nutrition in the school food environment and was developed based on the Model Bill Project by the Brazilian Institute for Consumer Defense which is aligned with Decree No 11,821/2023, since the decree was constructed using this bill as a reference. The criteria cover the following domains: (1) Food and Nutrition Education; (2) Food Distribution and Commercialization; (3) Marketing and Communication Strategies; and (4) Excellence Points: supervision and social control, coverage of private schools, regulatory strength (law or law regulated by decree), and prohibition of ultra-processed foods.

Based on the score obtained, measures were classified into two categories: (a) 0–7 points: regulations exist but need improvement or partially fulfill their function of promoting sustainable, adequate, and healthy nutrition in the school food environment; (b) 8–12 points: regulations effectively fulfill their role in promoting sustainable, adequate, and healthy nutrition in the school food environment [19]. Regulatory measures with a score ≥ 8 were considered high-quality.

### 2.3. Variables

This study’s outcome variables were assessed according to the strategic axes of Decree No. 11,821/2023: Nutrition Education (EAN; whether actions to promote healthy eating are implemented); food commercialization (whether the sale of foods is prohibited, healthiness index, number of UMPCP and UpCP sold, and whether the average variety of UpCP exceeds that of UMPCP); and marketing communication (presence of advertising strategies for selling UMPCP and UpCP). The implementation of healthy eating actions and the prohibition of food sales were assessed through the questions: “Does the canteen implement actions promoting healthy eating?” and “Does the school administration prohibit the sale of any type of food/product in the canteen?”, respectively. The total number of each subgroup (UMPCP and UpCP) sold was obtained by adding the items sold (ranging from 0 to 21 for UMPCP and 0 to 29 for UpCP). The Healthiness Index (HI) was calculated according to Tavares et al. (2021) [26] and reflects a score based on the presence of UMPCP subgroups and the absence of UpCP subgroups among those investigated. The closer the score is to 100%, the healthier the establishment, indicating a greater availability of healthy foods and lower availability of unhealthy foods. The variety of each subgroup (UMPCP and UpCP) sold was calculated as the mean number of different items, considering various flavors and brands. Subsequently, each canteen was categorized based on whether the average variety of UpCP exceeded that of UMPCP (yes/no). Finally, each canteen was categorized according to whether it implemented at least one of the following advertising strategies for selling UMPCP and UpCP (yes/no): supplier banner/poster, establishment banner/poster, clothing, product replica, menu, packaging, panel/TV, folder, displays, promotional gifts, or school/canteen app.

The explanatory variable of the study was the presence and quality of school food environment regulatory measures, assessing whether canteens were located in areas with existing municipal and/or state regulations covering private schools. When present, the quality was evaluated according to the criteria proposed by Rocha et al. (2023) [19]. Accordingly, each canteen was classified into three categories: located in areas with no school food environment regulations; presence of at least one regulation with a score < 8; or presence of at least one high-quality regulation (score ≥ 8).

### 2.4. Data Analysis

Descriptive analysis included the calculation of absolute and relative frequencies for categorical variables and means for quantitative variables, along with their 95% confidence intervals (95% CI). Crude and adjusted generalized linear models (GLM) were used to analyze the association between the presence and quality of regulations (explanatory variable) and the study’s quantitative outcomes (number of UMPCP and UpCP sold and Healthiness Index). Unstandardized beta coefficients (β) and their respective 95% CIs were calculated to estimate the effect size. Crude and adjusted binary logistic regression models were performed to analyze the association between the presence and quality of regulations (explanatory variable) and the study’s categorical outcomes (implementation of food and nutrition education actions, prohibition of the sale of certain foods, whether the average variety of UpCP exceeded UMPCP, and presence of UMPCP and UpCP advertising). Odds ratios (OR) and their respective 95% CIs were calculated to estimate effect size. All regression models were adjusted for the presence of a nutritionist, type of administration, and the number of customers and staff in the canteen. Data were analyzed using Stata version 17.0, with a significance level set at 5%.

### 2.5. Ethical Aspects

The study adhered to the ethical principles of the Declaration of Helsinki and Brazilian National Health Council Resolutions 466/2012 and 510/2016. Approval was obtained from the Ethics Committees of participating universities. Canteen managers who agreed to participate provided written informed consent.

## 3. Results

A total of 2241 school canteens from private schools located in the 27 Brazilian state capitals were evaluated. Further details of the regulatory measures for each municipal and state are presented in Tables S2 and S3 of the Supplementary Materials, respectively.

Table 1 presents the characterization of the canteens according to the presence and quality of municipal and state regulations. Approximately 41% of the canteens were located in areas with municipal regulations, with a similar percentage covered by state regulations. However, half of the canteens (51%) were located in municipalities or states without any current regulatory measures, and only 17.8% were covered by high-quality regulations (score ≥ 8).

Among canteens located in areas with municipal regulations, 71% addressed aspects of food distribution and commercialization, while only 26% included specific measures for Food and Nutrition Education actions and 24% explicitly addressed marketing communication. At the state level, 86% of regulations addressed food commercialization, 35% included food and nutrition education actions, and 28% restricted marketing communication. The proportion of measures regulated by decree was low at both municipal (26.4%) and state levels (19.6%).

Table 2 describes the administrative and operational characteristics of the canteens. Approximately 44.2% served up to 100 customers per day, and most had outsourced management (55.1%), fewer than five employees (77.2%), and a nutritionist on staff (50.6%). Only 27% of the canteens implemented nutrition education actions, and 32.8% prohibited the sale of certain types of foods. The mean Healthiness Index was 56.9. Regarding commercialization, a higher mean number of ultra-processed foods UpCP (6.58) was observed compared to unprocessed/minimally processed/processed foods UMPCP (6.03), and 65.8% of canteens had a greater variety of UpCP. Advertising strategies were observed in 74% of the canteens for both UpCP and UMPCP.

In the logistic regression analysis (Table 3), a positive association was observed between the presence of regulations, especially high-quality regulations, and the adoption of healthier practices in the canteens. Compared to canteens located in areas without regulations, those in areas with lower-quality regulations (<8 points) had 1.75 times higher odds of implementing food and nutrition education actions and 1.51 times higher odds of prohibiting the sale of certain foods. Among canteens in areas with higher-quality regulations (≥8 points), these odds increased to 1.73 and 2.49, respectively. Furthermore, the presence of regulations was associated with lower odds of offering a greater variety of AUPP (a reduction of 31% for lower-quality regulations and 36% for high-quality regulations).

The presence of advertising strategies was significantly lower only in canteens located in areas with high-quality regulations, with a 40% and 38% reduction in the odds of displaying UMPCP and UpCP advertising, respectively, compared to canteens without regulations.

In the generalized linear models (Table 4), the presence of regulations was also associated with better commercialization indicators. Canteens located in municipalities and states with regulations showed a higher Healthiness Index compared to those without regulations, with this association being more pronounced for high-quality regulations (β = 5.60; 95% CI: 4.62–6.58). The number of UMPCP sold was significantly higher in canteens located in areas with high-quality regulations (β = 1.54; 95% CI: 1.17–1.92), while the number of UpCP sold was lower in regulated areas, with the greatest reduction observed in high-quality regulations (β = −1.25; 95% CI: −1.71–−0.79).

Overall, the results demonstrate that the presence and especially the quality of regulatory measures is strongly associated with healthier school environments, characterized by a greater implementation of educational actions, restrictions on the sale of unhealthy foods, reduced advertising, and increased availability of unprocessed or minimally processed foods.

## 4. Discussion

The results of this study highlight the relevance of regulatory measures as instruments for promoting adequate and healthy eating in Brazilian private schools. Although normative coverage remains limited, with half of the canteens located in areas without regulations, the existence—and especially the quality—of regulations is positively associated with the adoption of healthier practices, including the implementation of Food and Nutrition Education actions, restrictions on the sale of unhealthy foods, and higher healthiness index in canteens. These findings reinforce the role of well-structured public policies in transforming school food environments.

The low coverage of regulatory measures identified in this study reflects regional inequalities and gaps in the implementation of school food policies in the private sector. Less than one-fifth of canteens are located in areas with high-quality regulations, and a considerable portion of existing laws lack decree-based regulation, limiting their practical applicability. As noted by Oguisso and Schmidt (1999) [27] and De Carvalho (2002) [28], regulation via decree is essential to ensure the implementation of laws and their continuity amidst government changes. Similarly, Rocha et al. (2023) [19] emphasize that decrees strengthen normative effectiveness and confer greater stability to public policies.

This study shows that canteens in municipalities or states with regulatory measures were more likely to implement food and nutrition education actions, especially when regulations were of high quality. This result aligns with previous evidence pointing to the importance of legal instruments in promoting educational practices [29,30]. By integrating EAN into the school routine, it contributes to the formation of healthy eating habits and engages the school community in sustainable dietary practices [31]. Studies indicate that well-structured educational actions reduce ultraprocessed food consumption and increase the acceptance of unprocessed foods [32,33,34]. Law No. 13,666/2018 [35] reinforces this role by including food and nutrition education in the school curriculum, making regulatory devices even more strategic.

The findings also indicated that the existence of regulations, particularly high-quality ones, is associated with higher availability of UMPCP, lower availability and variety of UpCP, and a higher healthiness index. These results are consistent with national and international studies demonstrating the potential of regulatory policies to modify food availability in school settings [21,36,37]. For instance, in Florianópolis, the implementation of the State Law regulating Santa Catarina school canteens reduced the sale of prohibited products, although full compliance remained challenging [36]. Similarly, Azeredo et al. (2020) [21] observed lower availability of soft drinks in schools located in states with restrictive legislation, although adherence was lower in private schools, highlighting the importance of monitoring mechanisms and manager engagement. Even with the legislation in place, some schools still showed limited levels of adherence, especially among private institutions and one of the factors affecting this situation is the lack of continuous monitoring and systematic evaluation of innovative measures [21].

International experiences also support these findings. In Boston (USA), regulation of sugary drinks resulted in a significant reduction in sales and consumption among students, attributed to complementary monitoring and awareness measures [37]. In Australia, policies categorizing foods as green, amber, and red based on healthiness promoted substantial improvements in food availability, with over 80% of items classified as healthy after a decade of implementation [16,17]. These examples underscore that robust regulations, accompanied by educational actions and monitoring mechanisms, are essential for policy effectiveness.

The low average healthiness index, along with the high availability, variety, and advertising strategies for UpCP, reveal the need to improve the quality of foods offered in canteens. In this context, the association of higher-quality regulations with a higher healthiness index is promising, reinforcing the role of well-structured public policies in promoting healthier school environments. Supporting this perspective, Gabriel et al. (2012) [38] analyzed legal instruments regulating the sale of food and beverages in Brazilian schools and emphasized that such regulations are fundamental to encourage healthy eating habits and prevent childhood obesity.

The analysis highlighted that marketing communication remains the least regulated area in Brazil, with more than half of the canteens located in areas without explicit restrictions on food advertising. However, canteens in areas with high-quality regulations were less likely to adopt advertising strategies for both UpCP and UMPCP. This relationship demonstrates the potential of well-structured legal instruments to curb marketing practices targeting children and adolescents. International reviews show that children do not have full cognitive capacity to distinguish advertising content from entertainment, making them more vulnerable to commercial persuasion [39]. Furthermore, exposure to ultraprocessed food advertising has been linked to increased consumption and poorer nutritional outcomes [40].

It is essential that regulations clearly specify which marketing practices are prohibited in school settings, including promotional materials, sponsorships, giveaways, apps, and indirect advertising. Normative clarity facilitates monitoring and reduces interpretive loopholes [41]. Nevertheless, few municipalities explicitly address monitoring and social control mechanisms, limiting compliance. National studies indicate that the lack of oversight is the main factor for noncompliance with school food laws [36,42]. Therefore, beyond creating regulations, strengthening monitoring and social participation mechanisms is imperative.

This study reinforces the importance of high-quality regulatory measures in promoting healthy school environments. However, less than 20% of canteens are covered by robust regulations, highlighting institutional fragility. Rocha et al. (2023) [19] identified that, in 2021, only 13.9% of Brazilian state and municipal regulations achieved scores considered high quality, with most not aligned with the Brazilian Dietary Guidelines. These findings align with the current study and indicate the need to strengthen coordination among federal, state, and municipal levels to expand coverage and effectiveness of regulations.

Decree No. 11,821/2023 represents a milestone in this process by establishing national guidelines and encouraging the development of local regulations based on scientific evidence. Recent initiatives, such as the National Strategy for Food and Nutrition Security in Cities, support the implementation of the decree in partnership with universities and civil society organizations [43]. This intersectoral coordination is essential for Brazil to advance in consolidating sustainable and equitable public policies promoting healthy school food environments.

This study’s strengths include the novelty of the approach, a representative sample of schools across all 27 capitals, and the simultaneous evaluation of different regulatory dimensions (food and nutrition education, commercialization, and marketing communication). Limitations include the potential that not all current regulatory measures were identified due to the absence of a centralized official database and differences in information availability on government websites. This study also did not assess the impact of regulatory measures at the individual level, such as students’ food consumption. A recent national study [44] addressed this issue, but only analyzed the presence of legislation, without considering its quality. The results indicated that the presence of the law was associated with lower consumption of ultra-processed foods among adolescents. Another limitation is that the timing of regulatory approval in each location did not coincide with the evaluation of the food environment. In other words, there is variation in the time it took for regulatory acts to be enacted across cities, which may have influenced the results. For example, Porto Alegre recently approved a law closely aligned with Decree No. 11,821/2023 and currently has the highest healthiness index among the capitals. However, when the food environment was reassessed after a longer period following the law’s approval, the healthiness index had decreased, although it remained higher compared to schools in other capitals [45]. Furthermore, it is important to note that these findings apply specifically to private schools, and their implications for public schools should be interpreted with caution or supported by additional evidence. Nevertheless, the study provides an updated and comprehensive overview of school food environment regulations in the country.

## 5. Conclusions

This study revealed a challenging scenario for the Brazilian school food environment in private schools, marked by the predominance of ultraprocessed foods and limited coverage of high-quality regulatory measures. Although the mere existence of regulations is associated with healthier practices, the quality of the regulations proved decisive for creating school food environments that are protective and health-promoting. Canteens located in municipalities and states with high-quality regulations were more likely to implement food and nutrition education actions, restrict the sale of unhealthy products, achieve a higher healthiness index, and use fewer ultraprocessed food advertising strategies. These results confirm the structuring role of well-formulated and enforced public policies in promoting adequate and healthy eating among children and adolescents. The data suggest that the presence of higher-quality laws favors healthier school food environments; however, it is necessary to strengthen community awareness and monitoring actions to ensure greater effectiveness of these laws. Expanding and strengthening local regulations, in line with Decree No. 11,821/2023, is essential to consolidate healthy school environments in private schools nationwide. Beyond establishing rules, it is necessary to invest in monitoring mechanisms, social control, and intersectoral training to ensure effective implementation and compliance with legislation. The findings of this study provide scientific evidence to support public managers and policymakers in developing more effective and equitable regulatory instruments aligned with the Brazilian Dietary Guidelines.

## Figures and Tables

**Table 1:** Characterization of school canteens according to the presence and quality of municipal and state regulations. Food sale in Brazilian Schools (Caeb) 2022–2024.

**Municipal regulatory measures**		
	**N (2241)**	**%/Mean (95% CI)**
**Has municipal regulation covering private schools**	913	40.7 (38.7–42.7)
**Food and nutrition education**		
Not mentioned in the regulatory measure	310	34.0(30.9–37.0)
Mentioned in the regulatory measure without provisions for its development	362	39.6 (36.5–42.8)
Mentioned in the regulatory measure and provides for its development	241	26.4 (23.5–29.3)
**Distribution and marketing of foods**		
No regulation of food distribution and marketing is mentioned	56	6.1 (4.6–7.7)
Regulation of food distribution and marketing is mentioned without distinguishing which foods are prohibited or allowed	209	22.9 (20.2–25.6)
Regulation of food distribution and marketing in the school environment is mentioned, distinguishing which foods are prohibited or allowed	648	71.0 (68.0–73.9)
**Marketing communication**		
No regulation of marketing communication is mentioned	515	56.4 (53.2–59.6)
Marketing communication is prohibited	179	19.6 (17.0–22.2)
Marketing communication is prohibited regarding foods banned in the school environment by regulatory measure, and describes the prohibited resources.	219	24.0 (21.2–26.8)
**The regulatory measure provides for monitoring and social control**	351	38.4 (35.2–41.6)
**The regulatory measure is a law**	913	100
**The regulatory measure is a law e está regulamentada por um decreto**	241	26.4 (23.5–23.3)
**The regulatory measure prohibits ultra-processed foods**	0	0
**Score of municipal regulations**		
0 to 7 points	515	56.4 (53.1–59.6)
8 to 12 points	398	43.6 (40.3–46.8)
Average total score	913	6.2 (5.9–6.3)
**State regulatory measures**		
**Has municipal regulation covering private schools**	911	40.7 (38.1–42.6)
**Food and nutrition education**		
Not mentioned in the regulatory measure	292	32.1 (29.0–35.0)
Mentioned in the regulatory measure without provisions for its development	302	33.1 (30.0–36.2)
Mentioned in the regulatory measure and provides for its development	317	34.8 (31.7–37.9)
**Distribution and marketing of foods**		
No regulation of food distribution and marketing is mentioned	126	13.8 (22.6–16.0)
Regulation of food distribution and marketing is mentioned without distinguishing which foods are prohibited or allowed	0	0
Regulation of food distribution and marketing in the school environment is mentioned, distinguishing which foods are prohibited or allowed	785	86.2 (83.9–99.4)
**Marketing communication**		
No regulation of marketing communication is mentioned	475	52.1 (48.9–55.4)
Marketing communication is prohibited	179	19.7 (17.0–22.2)
Marketing communication is prohibited dos alimentos proibidos no ambiente escolar pela medida regulatória, e descreve os recursos proibidos	257	28.2 (25.2–31.1)
**The regulatory measure provides for monitoring and social control**	638	70 (67.0–73.0)
**The regulatory measure covers private schools**	911	100
**The regulatory measure is a law**	911	100
**The regulatory measure is a law e está regulamentada por um decreto**	179	19.6 (17.0–22.2)
**The regulatory measure prohibits ultra-processed foods**	108	11.9(1.0–14.0)
**Total score of state regulations**		
0 to 7 points	672	73.8 (70.9–76.6)
8 to 12 points	239	26.2 (23.3–29.1)
Average total score	911	6.7 (6.6–6.8)
**Existence and quality of the regulatory measure**		
No regulation of the school food environment	1144	51.0 (49.0–53.1)
Has at least one municipal and/or state regulation, but with a score < 8	699	31.2 (29.3–33.1)
Has at least one municipal and/or state regulation, with a score ≥ 8	398	17.8 (16.2–19.3)

**Table 2:** Characterization of school canteens, according to administrative aspects and variables related to food and nutrition education actions, marketing and communication of food products. Food sale in Brazilian schools (Caeb), 2022–2024.

Variables	N	%/Mean (95% CI)
**Administrative variables**		
**Average number of customers served per day**		
*Up to 100 customers*	991	44.2 (42.1–46.2)
*101 to 200 customers*	571	25.5 (23.6–27.8)
*More than 200 customers*	679	30.3 (28.3–32.2)
**Type of management**		
*Outsourced*	1236	55.1 (53.0–57.2)
*Owned*	869	38.8 (36.7–40.7)
*Parents and teachers association or others*	136	6.1 (5.0–7.5)
**Number of employees**		
*<5 employees*	1730	77.2 (75.4–78.9)
*5 to 10 employees*	474	21.1 (19.4–22.8)
*11 or more employees*	37	1.7 (1.1–2.1)
Presence of a nutritionist	1133	50.6 (48.4–52.6)
**Variables of the Food and Nutrition Education Axis**		
Develops actions that encourage healthy eating	604	27.0 (25.1–28.7)
**Variables of the Sale Axis**		
Existence of a prohibition by the school management on the sale of any type of food/product in the canteen	736	32.8 (30.8–34.7)
Total number of UMPCP sold	2241	6.03 (5.9–6.2)
Total number of UpCP sold	2241	6.6 (6.4–6.7)
Healthiness index	2241	56.9 (56.5–57.3)
Average variety of UpCP is greater than UMPCP	1429	65.8 (63.7–67.7)
**Variables of the Marketing Communication Axis**		
Has at least one advertising strategy for UMPCP sold	1658	74.0 (72.1–75.8)
Has at least one advertising strategy for UpCP sold	1638	73.1 (71.2–74.9)

**Table 3:** Crude and adjusted Binary Logistic Regression models of the association between the presence and quality of municipal and/or state regulations with outcome variables related to food and nutrition education actions, marketing and marketing communication of food in school canteens. Food sale in Brazilian schools (Caeb), 2022–2024.

Dependent Variables/Explanatory Variable	Crude OR	95% CI	*p*-Value	Adjusted OR *	95% CI	*p*-Value
**Implementation of Food and Nutrition Education actions**						
**Score of municipal and/or state regulation**						
No regulation of the school food environment	Reference	-	-	Reference	-	-
Has at least one municipal and/or state regulation, but with a score < 8	1.68	1.35; 2.08	<0.001	1.75	1.38; 2.22	<0.001
Has at least one municipal and/or state regulation, with a score ≥ 8	2.65	2.07; 3.39	<0.001	1.73	1.33; 2.26	<0.001
**Prohibition of any type of food**						
**Score of municipal and/or state regulation**						
No regulation of the school food environment	Reference	-	-	Reference	-	-
Has at least one municipal and/or state regulation, but with a score < 8	1.56	1.27; 1.91	<0.001	1.51	1.21; 1.88	<0.001
Has at least one municipal and/or state regulation, with a score ≥ 8	3.30	2.60; 4.18	<0.001	2.49	1.94; 3.21	<0.001
**Average variety of UpCP sold is greater than UMPCP**						
**Score of municipal and/or state regulation**						
No regulation of the school food environment	Reference	-	-	Reference	-	-
Has at least one municipal and/or state regulation, but with a score < 8	0.61	0.50; 0.74	<0.001	0.69	0.56; 0.86	0.001
Has at least one municipal and/or state regulation, with a score ≥ 8	0.54	0.42; 0.68	<0.001	0.64	0.49; 0.82	0.001
**Has at least one advertising strategy for UMPCP**						
**Score of municipal and/or state regulation**						
No regulation of the school food environment	Reference	-	-	Reference	-	-
Has at least one municipal and/or state regulation, but with a score < 8	1.09	0.88; 1.35	0.425	0.90	0.71; 1.14	0.403
Has at least one municipal and/or state regulation, with a score ≥ 8	0.88	0.69; 1.14	0.359	0.60	0.45; 0.80	0.001
**Has at least one advertising strategy for UpCP**						
**Score of municipal and/or state regulation**						
No regulation of the school food environment	Reference	-	-	Reference	-	-
Has at least one municipal and/or state regulation, but with a score < 8	1.09	0.80; 1.35	0.425	0.97	0.77; 1.22	0.799
Has at least one municipal and/or state regulation, with a score ≥ 8	0.88	0.68; 1.14	0.359	0.62	0.47; 0.81	0.001

* Adjusted for number of clients served, type of administration, number of employees and presence of a nutritionist. Note: UMPCP: Unprocessed, minimally processed or processed foods and organic culinary products based on these foods; UpCP: Ultra-processed foods and organic products based on these foods; CI: Confidence Interval; OR: Odds Ratio.

**Table 4:** Crude and adjusted generalized linear models of the association between the presence and quality of municipal and/or state regulations and outcome variables related to food marketing actions in school canteens. Food sale in Brazilian schools. (Caeb), 2022–2024.

Dependent Variables/Explanatory Variables	Crude β	95% CI	*p*-Value	Adjusted β *	95% CI	*p*-Value
**Healthiness index**						
**Score of municipal and/or state regulation**						
No regulation of the school food environment	Reference	-	-	Reference	-	-
Has at least one municipal and/or state regulation, but with a score < 8	2.81	1.95; 3.68	<0.001	1.59	0.78; 2.41	<0.001
Has at least one **municipal** and/or state regulation, with a score ≥ 8	7.71	6.67; 8.76	<0.001	5.60	4.62; 6.58	<0.001
**Total number of UMPCP sold**						
**Score of municipal and/or state regulation**						
No regulation of the school food environment	Reference	-	-	Reference	-	-
Has at least one municipal and/or state regulation, but with a score < 8	0.57	0.24; 0.90	0.001	0.15	−0.15; 0.46	0.321
Has at least one municipal and/or state regulation, with a score ≥ 8	2.50	2.10; 2.90	<0.001	1.54	1.17; 1.92	<0.001
**Total number of UpCP sold**						
**Score of municipal and/or state regulation**						
No regulation of the school food environment	Reference	-	-	Reference	-	-
Has at least one municipal and/or state regulation, but with a score < 8	−0.83	−1.21; −0.45	<0.001	−0.64	−1.02; −0.25	0.001
Has at least one municipal and/or state regulation, with a score ≥ 8	−1.35	−1.81; −0.89	<0.001	−1.25	−1.71; −0.79	<0.001

* Adjusted for number of clients served, type of administration, number of employees and presence of a nutritionist.

## Data Availability

The original contributions presented in this study are included in the article/Supplementary Materials. Further inquiries can be directed to the corresponding author.

## Supplementary Materials

The following supporting information can be downloaded at: https://www.mdpi.com/article/10.3390/ijerph23020244/s1, Table S1: Classification of foods sold in private school canteens, according to the NOVA classification, adopted by the Dietary Guidelines for the Brazilian Population; Table S2: Municipal regulatory measures and their respective scores; Table S3: State regulatory measures and their respective scores.
