# Supplementary material for: Do Higher-Quality Regulatory Measures Promote a Healthier School Food Environment?
Source: Int J Environ Res Public Health. 2026 Feb 14;23(2):244. doi: 10.3390/ijerph23020244 (PMC12940654; doi:10.3390/ijerph23020244)
Supplement: Supplementary file 1 [file ijerph-23-00244-s001.zip › ijerph-4115527-supplementary.pdf]

SUPPLEMENTARY MATERIAL

Table S1. - Classification of foods sold in private school canteens, according to the NOVA classification, adopted by the Dietary Guidelines for the Brazilian Population.

Food groups	List of foods evaluated
**UMPCP (21 items)**	Mineral water (with or without gas); coconut water; natural fruit juice; 100% natural juice from a carton, can, or bottle; coffee, natural tea; milk smoothie with fruit/vitamin; fresh fruit; dried/dehydrated fruit; simple fruit salad; açaí without sugar or syrup; homemade cake; sweet made with fruits or vegetables; homemade biscuit; cheese bread (homemade); tapioca without ultra-processed filling; pizza without ultra-processed filling; baked savory snack without ultra-processed filling; fried savory snack without ultra-processed filling; sandwich without ultra-processed filling; popcorn made with natural sweet or salty grains.
**UpCP (29 items)**	Refreshments; fruit nectar in cartons, cans, or bottles; ready-to-drink tea; flavored milk drinks and yogurt; soy-based drinks; regular soda; zero-calorie, light, or diet soda; energy drinks; isotonic drinks; fruit salad with toppings; açaí with sugar or syrup; açaí with toppings; cereal bars; breakfast cereal; ultra-processed cakes; sweets with ultra-processed ingredients; bonbons or chocolate; treats; popsicles or ice cream; sweet biscuits with or without filling; packaged snacks, chips, savory biscuits/cookies; frozen cheese bread or mix; tapioca with ultra-processed filling; pizza with ultra-processed filling; baked savory snack with ultra-processed filling; fried savory snack with ultra-processed filling; sandwich with ultra-processed filling; ultra-processed popcorn; packaged popcorn, sweet and/or salty.

Table S2. - Municipal regulatory measures and their respective scores.

Municipality	Has school environment regulation	Title	Date	Description	Scope	Acess link	Final score
Rio Branco (AC)	NO	-	-	-	-	-	-

Maceió (AL)	NO	-	-	-	-	-	-
Manaus (AM)	YES	Law No. 1.414	22/01/2010	Prohibits the commercialization, distribution, and advertising of foods, soft drinks, and industrialized juices that contain harmful substances or high levels of trans fat, free sugar, and salt. Provides for teacher training in nutrition education. Applies to both public and private schools.	Public/Private	https://goo.gl/9W56pg	10
		Decree No. 741/11	12/01/2011	Regulates Law No. 1,414 of January 22, 2010, concerning healthy eating in public and private schools in Manaus.	Public/Private	https://www.legisweb.com.br/legislacao/?id=175982	
Macapá (AP)	NO	-	-	-	-	-	-
Salvador (BA)	YES	Law No. 8,292	16/05/2012	Prohibits the sale of listed unhealthy foods (including soft drinks, candies, and snacks, among others), requires the daily offering of at least two fruit varieties, and mandates the display of educational materials on healthy eating. Applies to both public and private schools.	Public/Private	https://goo.gl/NDvF6T	4
Fortaleza (CE)	YES	Law No. 8.824	30/03/2004	Establishes criteria for granting authorization for food and beverage sales in school canteens in Fortaleza and provides other measures.	Public/Private	https://leismunicipais.com.br/a/ce/f/fortaleza/lei-ordinaria/2004/882/8824/lei-ordinaria-n-8824-2004-dispoe-sobre-os-criterios-de-concessao-de-alimentos-e-bebidas-nas-cantinas-das-escolas-de-fortaleza-e-da-outras-providencias	3

Brasília (DF)	YES	Law No. 5.146	19/08/2013	Prohibits the commercialization and advertising of listed unhealthy products (including soft drinks, candies, and fried foods, among others) and requires the daily offer of at least one fruit variety. Applies to both public and private schools.	Public/Private	https://legislacao.cl.df.gov.br/Legislacao/buscarLei-10674!buscarLei.action	10
		Decree No. 36900	23/11/2015	Regulates Law 5,146/13, which prohibits the sale and advertising of listed unhealthy products (including soft drinks, candies, and fried foods, among others) and requires the daily offer of at least one fruit variety.	Public/Private	https://goo.gl/UFjNgr	
		Decree No. 37346	17/05/2016	Amends §2 of Art. 3 of Decree No. 36,900 of November 23, 2015, which regulates Law No. 5,146 of August 19, 2013, establishing guidelines for promoting adequate and healthy eating in the Federal District's school network.	Public/Private	http://www.sinj.df.gov.br/sinj/Norma/14d039923cf848ac920cd005ea29954d/Decreto_37346_17_05_2016.html	
		Law No. 3.695	08/11/2005	Prohibits the sale and advertising of listed unhealthy products (including soft drinks, candies, and fried foods, among others), requires the daily offer of at least one fruit variety, and provides for teacher training in nutrition education. Applies to both public and private schools.	Public/Private	http://www.sinj.df.gov.br/sinj/Norma/51622/Lei_3695_08_11_2005.html	
Vitória (ES)	YES	Law No. 8106	25/11/2011	Law No. 8,106 does not mention canteens but requires public and private schools to offer 90% of meals as healthy options. Industrialized foods with high calories and low nutrients are completely prohibited.	Public/Private	https://www.legisweb.com.br/legislacao/?id=127267	4
		Law No. 6.786	24/11/2006	Prohibits the sale of fried foods, sweets, and similar items in school meals and canteens within the municipal school network.	Public	https://goo.gl/abZKSd	

Goiânia (GO)	NO	-	-	-	-	-	-
São Luís (MA)	NO	-	-	-	-	-	-
Belo Horizonte (MG)	YES	Law No. 8.650	25/09 /2003	Prohibits the commercialization, acquisition, preparation, distribution, and advertising of listed unhealthy products (alcoholic beverages, candies, and similar items). Applies to both public and private schools.	Public/Private	https://goo.gl/9dDwJU	4
Campo Grande (MS)	YES	Law No. 4.992	30/09 /2011	Prohibits the sale and advertising of listed unhealthy foods (including soft drinks, candies, and fried snacks, among others) and requires the daily offering of at least one fruit variety. Applies to both public and private schools.	Public/Private	https://www.normasbrasil.com.br/norma/lei-4992-2011-campo-grande_172949.html	8
Cuibá (MT)	YES	Law No 4.382	17/07 /2003	Establishes criteria for granting snack and beverage service concessions in educational units located in the municipality of Cuiabá.	Public/Private	https://cm-cuiaba-mt.jusbrasil.com.br/legislacao/573739/lei-4382-03	5
		Law No. 4.589	03/06 /2004	Amends and updates provisions of Law No. 4,382 of July 17, 2003. Explicitly prohibits the sale of: (I) alcoholic beverages; (II) candies, lollipops, and chewing gum; (III) fried snacks; and (IV) industrialized popcorn.	Public/Private	https://cm-cuiaba-mt.jusbrasil.com.br/legislacao/571864/lei-4589-04	
Belém (PA)	NO	-	-	-	-	-	-
João Pessoa (PB)	NO	-	-	-	-	-	-
Recife (PE)	NO	-	-	-	-	-	-
Teresina (PI)	YES	Law No. 5380	28/05 /2019	Requires menus in the establishments mentioned to include information about the presence or absence of gluten, lactose, or sugar, as well as whether the foods are diet or light.	Public/Private	https://www.normasbrasil.com.br/norma/lei-5380-2019-teresina_378612.html	2

Curitiba (PR)	YES	Law No. 10.950	03/05/2004	Regulates the foods and beverages to be sold in school canteens located in the municipality of Curitiba and provides related measures.	Public/Private	https://cm-curitiba.jusbrasil.com.br/legislacao/339198/lei-10950-04	4
		Law No. 11.299	16/12/2004	Establishes the Municipal Policy for the Prevention of Childhood Obesity and requires that meal menus served to children and adolescents be prepared by a nutritionist from the municipal staff, in collaboration with the Municipal School Food Council.	Public	https://leismunicipais.com.br/a/pr/c/curitiba/lei-ordinaria/2004/1130/11299/lei-ordinaria-n-11299-2004-cria-o-programa-municipal-de-prevencao-e-controle-da-obesidade-em-criancas-e-adolescentes-em-curitiba-e-da-outras-providencias?q=obesidade+infantil	
Rio de Janeiro (RJ)	YES	Decree No. 21.217	01/04/2002	Prohibits the acquisition, preparation, and distribution of listed unhealthy products (including candies and powdered drink mixes, among others) and establishes a nutrition education program. Applies to the municipal school network.	Public	http://www.rio.rj.gov.br/dlstatic/10112/5118607/4132721/Decreton21.217de1.deabrilde2002.pdf	9
		Law No. 7988	12/07/2023	Establishes actions to combat childhood obesity by promoting healthy environments in public and private schools in the municipality of Rio de Janeiro.	Public/Private	https://mail.camara.rj.gov.br/APL/Legislativos/contlei.nsf/c5e78996b82f9e0303257960005fdc93/9c16558886a2a7f5032589e9004e4e25?OpenDocument	

		Decree No. 52842	11/07 /2023	Regulates Municipal Law No. 7,987 of July 11, 2023, which establishes actions to combat childhood obesity and provides other measures.	Public/Private	https://doweb.rio.rj.gov.br/apifront/portal/edicoes/imprimir_materia/965916/5930#:~:text=DECRETA%3A,Par%C3%A1grafo%20%C3%BAnico.	
Natal (RN)	NO	-	-	-	-	-	-
Porto Velho (RO)	YES	Law No. 2.306	07/06 /2016	Establishes guidelines, objectives, and actions for the implementation of the Municipal School Feeding Policy and provides other measures.	Public	https://leismunicipais.com.br/a/ro/p/porto-velho/lei-ordinaria/2016/231/2306/lei-ordinaria-n-2306-2016-estabelece-diretrizes-objetivos-e-acoes-para-a-instituicao-da-politica-municipal-de-alimentacao-escolar-e-da-outras-providencias?q=alimenta%C3%A7%C3%A3o+escolar	3
Boa vista (RR)	NO	-	-	-	-	-	-
Porto Alegre (RS)	YES	Law No. 10.167	24/01 /2007	Prohibits the sale and advertising of unhealthy products within the established parameters. Applies to both public and private schools.	Public/Private	https://goo.gl/euy92Q	8

Florianópolis (SC)	YES	Law No. 5.853	04/06/2001	Prohibits the sale of listed unhealthy products (including soft drinks, candies, and industrialized snacks, among others) and allows the sale of listed healthy foods (including bread, fruits, and natural juices). Requires the display of educational materials on healthy eating. Applies to both public and private schools..	Public/Private	https://goo.gl/b4KCDc	4
		Ordinance No. 221	16/12/2013	Regulates the trade, donation, and use of food within the premises of the Municipal Education Network.	Public	https://www.pmf.sc.gov.br/arquivos/arquivos/pdf/26_06_2014_10.14.21.a3104ead3a22c10124c3f49d3890977b.pdf	
Aracaju (SE)	YES	Law No. 3.814	14/01/2010	Prohibits the sale of listed unhealthy products (including soft drinks, candies, and snacks, among others) and promotes the sale of listed healthy foods (including fruits and natural juices, among others). Applies to both public and private schools.	Public/Private	https://leismunicipais.com.br/a/se/a/aracaju/lei-ordinaria/2010/382/3814/lei-ordinaria-n-3814-2010-dispoe-sobre-a-alimentacao-oferecida-nas-cantinas-e-lanchonetes-localizadas-nas-instituicoes-de-ensino-publicas-e-privadas-dentro-da-circunscricao-do-municipio-de-aracaju-e-da-outras-providencias?q=3814	5
São Paulo (SP)	NO	-	-	-	-	-	-

Palmas (TO)	YES	Law No.1.210	08/07/2003	Determines the food products that may be acquired by schools, establishes rules for their storage, and provides other technical guidelines related to school feeding. Applies to the municipal school network.	Public	https://goo.gl/YKcoZA	3

TABLE S3 - State regulatory measures and their respective scores.

State	Has school environment regulation	Title	Date	Description	Scope	Acess link	Final score
Alagoas	NO	-	-	-	-	-	-
Amazonas	YES	Law No. 4.325	05/06/2016	Prohibits the sale, acquisition, and distribution of products that contribute to childhood obesity in canteens and similar establishments located in public and private schools within the State of Amazonas.	Public/Private	https://legisla.imprensaoficial.am.gov.br/diario_am/12/2016/7/1358#:~:text=Art.,situadas%20no%20Estado%20do%20Amazonas.	7
Amapá	NO	-	-	-	-	-	-
Pará	NO	-	-	-	-	-	-
Goiás	YES	Law No. 16.333	26/08/2008	Establishes the State Policy for Food and Nutrition Education.	Public	https://legisla.casacivil.go.gov.br/api/v2/pesquisa/legislacoes/87050/pdf	3

Pernambuco	YES	Law No. 18.509	16/04/2024	Establishes the Assisted Balanced Diet Policy (PABA) in the educational institutions indicated and provides other measures.	Private	https://legis.alepe.pe.gov.br/texto.aspx?id=77198	4
Ceará	YES	Law No. 15.205	24/07/2012	Establishes the State "Healthy Canteen" Program in public schools of the State of Ceará, aiming to promote actions that encourage healthier eating habits in the school environment and combat obesity.	Public	https://belt.al.ce.gov.br/index.php/legislacao-do-ceara/organizacao-tematica/educacao/item/1858-lei-n-15-205-de-19-07-12-d-o-24-07-12	1
Tocantins	YES	Law No. 4501	11/07/2024	Establishes actions to combat childhood obesity within the State of Tocantins.	Public/Private	https://www.al.to.leg.br/arquivos/lei_4501-2024_70831.PDF	2
Rondônia	YES	Ordinance No. 1.851	25/09/2012	Prohibits the sale of listed unhealthy products (including candies and snacks, among others) and allows the sale of listed healthy products (including milk, fruits, and whole grains, among others). Prohibits canteens from operating during students' entry and exit times. Applies to the state education network.	Public	https://goo.gl/PPPU1X	2
Rio Grande do Norte	YES	Law No. 9.434	29/12/2010	Regulates the sale of food products by private individuals inside state schools in Rio Grande do Norte and provides other measures.	Public	https://www.normasbrasil.com.br/norma/lei-9434-2010-rn_152728.html	3
Piauí	YES	Law No. 7.028	22/08/2017	Establishes the obligation to inform consumers about the ingredients used in food served in restaurants, school canteens, hospitals, bakeries, ice cream shops, hotels, and similar	Public/Private	https://www.normasbrasil.com.br/norma/lei-7028-2017-pi_348142.html	5

				establishments, and provides other measures.			
		Normative Instruction GSE/ADM No. 005	14/03/2018	Prohibits the offering of low-nutritional beverages such as soft drinks, artificial refreshments, beverages or concentrates based on guaraná or currant syrup, ready-to-drink teas, and similar drinks, as well as fried snacks, savory chips, candies, sweets, and other foods with high levels of fats, salt, and sugar, and foods considered “empty calories.”	Public/Private	https://www.seduc.pi.gov.br/arquivos/normativas/normativa_1051793268.instrucao_normativa_005_funcionamento_cantina.pdf	
Espírito Santo	YES	Ordinance No 038R	14/04/2010	Prohibits the sale of listed unhealthy products (including soft drinks, candies, and industrialized snacks, among others) and allows the sale of listed healthy foods (including bread, fruits, and natural juices). Establishes that canteens serve only school staff. Applies to the state education network.	Public	http://sinepe-es.org.br/?37/noticia/portaria-no-038-r	3
Roraima	YES	Resolution CEAE No 1/	30/07/2012	Regulates snack services in public educational units that provide basic education in the state, which must comply with food and nutritional quality standards essential for students’ health.	Public	https://www.normasbrasil.com.br/norma/resolucao-1-2012-rr_243216.html	4

		No 2173					
Santa Catarina	YES	Law No. 12.061	18/12/2001	Prohibits the sale of listed unhealthy products (including soft drinks, candies, and industrialized snacks, among others) and allows the sale of listed healthy foods (including bread, fruits, and natural juices). Requires the display of educational materials on healthy eating. Applies to both public and private schools.	Public/Private	https://goo.gl/pa8N9L	4
Paraíba	YES	Law No. 10.431	20/01/2015	Prohibits the sale of soft drinks in public and private schools.	Public/Private	https://goo.gl/U1CFHb	4

Mato Grosso	YES	Law No. 8.681	13/07/2007	This law regulates the food offered in public and private schools that provide early childhood and basic education in the State of Mato Grosso.	Public/Private	https://leisestaduais.com.br/mt/lei-ordinaria-n-8681-2007-mato-grosso-disciplina-a-alimentacao-oferecida-nas-unidades-escolares-publicas-e-privadas-que-atendam-a-educacao-infantil-e-basica-do-estado-de-mato-grosso#:~:text=Disciplina%20a%20alimenta%C3%A7%C3%A3o%20oferecida%20nas,Autor%3A%20Deputado%20Otaviano%20Pivetta.	6
		Law No. 8.944	29/07/2008	Prohibits the sale of: soft drinks, chewing gum, and similar products; processed foods with high levels of saturated fats, trans fats, and salt; as well as fried snacks and foods with nutrients harmful to health, among others.	Public/Private	https://leisestaduais.com.br/mt/lei-ordinaria-n-8944-2008-mato-grosso-altera-a-redacao-do-art-2%C2%BA-da-lei-n%C2%BA-8681-de-13-de-julho-de-2007	
		Ordinary Law No. 11.831	18/07/2022	Establishes the Balanced Diet Program within the State of Mato Grosso.	Public/Private	https://leisestaduais.com.br/mt/lei-ordinaria-n-11831-2022-mato-grosso-institui-o-programa-de-alimentacao-balanceada-no-ambito-do-estado-de-mato-grosso?q=Educa%C3%A7%C3%A3o	

		Law No. 11.575	17/11/2021	The Program for the Prevention and Treatment of Childhood Obesity is hereby established in public and private educational institutions within the State of Mato Grosso.	Public	https://leisestaduais.com.br/mt/lei-ordinaria-n-11575-2021-mato-grosso-fica-instituido-o-programa-de-prevencao-e-tratamento-da-obesidade-infantil-nas-instituicoes-de-ensino-publicas-e-privadas-no-ambito-do-estado-de-mato-grosso?q=%22obesidade%20infantil%22	
São Paulo	YES	Joint Ordinance COGSP/CEI/DSE	23/03/2005	It prohibits the sale of products that may contribute to obesity, allowing only the listed healthy products (including fruits, cakes, and natural or pulp juices - among others). This applies to schools in the state education system.	Public	http://siau.edunet.sp.gov.br/ItemLise/arquivos/notas/portconj_cogsp_cei_dse(doe230305).htm	4
		Law No. 17.340	11/03/2021	This law prohibits the sale of processed foods containing trans fats in basic education schools.	Public/Private	https://www.al.sp.gov.br/repositorio/legislacao/lei/2021/lei-17340-11.03.2021.html	

Bahia	YES	Ordinary Law No. 14.045	27/12/2018	Amends Law No. 13.582/2016, to provide for advertising aimed at children in basic education establishments in the State of Bahia.	Public/Private	https://leisestaduais.com.br/ba/lei-ordinaria-n-14045-2018-bahia-altera-a-lei-no-13-582-2016-para-dispor-sobre-a-publicidade-infantil-nos-estabelecimentos-de-educacao-basica-no-estado-da-bahia	4
		Law No. 13.852	14/09/2016	It prohibits advertising of food and beverages aimed at children that are low in nutrients and high in sugar, saturated fat, and sodium.	Public/Private	https://goo.gl/4iPzTD	
Paraná	YES	Law No. 14.423	03/06/2004	Prohibits the sale and advertising of unhealthy foods listed in the legislation (including soft drinks, candies, and fried snacks, among others), mandates the daily offering of at least one variety of fruit, and the display of informational materials about healthy eating. It applies to both public and private schools.	Public/Private	https://goo.gl/mJybMS	6

		Law No. 16.085	17/04/2009	It stipulates that the establishments specified, which operate within private schools, are required to disclose the information mentioned regarding the presence and breakdown of quantities in their nutritional tables of the foods they sell.	Private	https://leisestaduais.com.br/pr/lei-ordinaria-n-16085-2009-parana-dispoe-que-os-estabelecimentos-que-especifica-que-funcionam-dentro-das-escolas-da-rede-particular-de-ensino-ficam-obrigados-a-divulgarem-informacoes-que-menciona-referentes-a-presenca-e-a-discriminacao-de-quantidades-em-suas-tabelas-nutricionais-dos-alimentos-comercializados#:~:text=DISP%C3%95E%20QUE%20OS%20ESTABELECIMENTOS%20QUE,TABELAS%20NUTRICIONAIS%20DOS%20ALIMENTOS%20COMERCIALIZADOS.	

		Law No. 14.855	19/10/2005	Prohibits the sale of listed unhealthy products (including soft drinks, candies, and processed snacks - among others) and allows the sale of listed healthy foods (including bread, fruits, and natural juices). It provides for the display of informational materials about healthy eating. It is valid for both public and private school systems.	Public/Private	https://leisestaduais.com.br/pr/lei-ordinaria-n-14855-2005-parana-dispoe-sobre-padroes-tecnicos-de-qualidade-nutricional-a-serem-seguidos-pelas-lanchonetes-e-similares-instaladas-nas-escolas-de-ensino-fundamental-e-medio-particulares-e-da-rede-publica	
		Law No. 7.085	20/10/2005	This law establishes technical standards for nutritional quality to be followed by cafeterias and similar establishments located in private and public elementary and secondary schools.	Public/Private	https://www.legislacao.pr.gov.br/legislacao/pesquisarAto.do?action=exibir&codAto=6351&indice=1&totalRegistros=1	
Mato Grosso do Sul	NO	-	-	-	-	-	-
Acre	YES	Law No. 3.134	01/06/2016	It mandates the display of educational materials that promote healthy eating and the training of faculty in nutritional education. It prohibits the display of posters that encourage the consumption of unhealthy products, such as chips, candies, and soft drinks – among others.	Public/Private	https://goo.gl/hkAbYD	6
Maranhão	YES	Law No. 10.342	20/10/2015	Establishes guidelines, objectives, and actions for the implementation of the state school food policy, and provides other measures.	Public/Private	http://stc.ma.gov.br/legisla-documento/?id=3988	6

		Law No. 11.196	19/12/2019	This law addresses the promotion of healthy eating and mandates the exclusion of ultra-processed and sugary foods in public and private schools within the state of Maranhão.	Public/Private	https://www.legisweb.com.br/legislacao/?id=387976	
Rio de Janeiro	YES	Law No. 9779	04/07/2022	Institutes the program for improving the quality of school meals in the state of Rio de Janeiro.	Public	https://leisestaduais.com.br/rj/lei-ordinaria-n-9779-2022-rio-de-janeiro-institui-o-programa-de-melhoria-na-qualidade-da-alimentacao-escolar-no-estado-do-rio-de-janeiro?q=ALIMENTA%C3%87%C3%83O%20ESCOLA	9
		Law No. 10233	11/12/2023	Provides for the creation of the program "brazilian dietary guidelines in schools," with the aim of promoting this official publication in the school environment and reducing the consumption of processed and ultra-processed foods in the state of Rio de Janeiro.	Public/Private	https://leisestaduais.com.br/rj/lei-ordinaria-n-10233-2023-rio-de-janeiro-dispoe-sobre-a-criacao-do-programa-guia-alimentar-para-a-populacao-brasileira-nas-escolas-com-o-intuito-de-promover-esta-publicacao-oficial-no-ambiente-escolar-e-reduzir-o-consumo-de-alimentos-processados-e-ultraprocessados-no-estado-do-rio-de-janeiro?q=ALIMENTA%C3%87%C3%83O%20ESCOLA	

		Law No. 4.508	11/1/2005	It is prohibited to sell, purchase, manufacture, and distribute products that contribute to childhood obesity in bars, canteens, and similar establishments located in public and private schools in the State of Rio de Janeiro.	Public/Private	https://leisestaduais.com.br/rj/lei-ordinaria-n-4508-2005-rio-de-janeiro-altera-a-lei-n-4508-de-11-de-janeiro-de-2005-e-da-outras-providencias	
Sergipe	YES	Law No. 8.178-A	21/12/2016	It prohibits the sale of products that contribute to childhood obesity in canteens and similar establishments located in public and private schools throughout the State of Sergipe.	Public/Private	https://www.legisweb.com.br/legislacao/?id=337364	7
Minas Gerais	YES	Law No. 15.072	05/04/2004	It prohibits the supply and sale of products and preparations with high levels of calories, saturated fat, trans fat, free sugar, and salt, or with few nutrients. It establishes a state-level nutritional education program. It is valid for both public and private school systems.	Public/Private	https://www.almg.gov.br/consulte/legislacao/completa/completa.html?tipo=LEI&num=15072&ano=2004	7
Rio Grande do Sul	YES	Law No. 15.216	30/07/2018	This law addresses the promotion of healthy eating and prohibits the sale of products that contribute to obesity, diabetes, and hypertension in canteens and similar establishments located in public and private schools in the State of Rio Grande do Sul.	Public/Private	https://www.normasbrasil.com.br/norma/lei-15216-2018-rs_365652.html	10

		Decree No. 54994	17/01/2020	Regulates Law No. 15,216, of July 30, 2018, which provides for the promotion of healthy eating and prohibits the sale of products that contribute to obesity, diabetes, and hypertension in canteens and similar establishments located in public and private schools in the State of Rio Grande do Sul.	Public/Private	http://www.al.rs.gov.br/filerepository/repLegis/arquivos/DEC_54.994.pdf	
Distrito Federal	YES	Law No. 6.475	03/01/2020	It prohibits the offering of processed meats in the school lunches served to students in public schools in the Federal District.	Public	http://www.sinj.df.gov.br/sinj/Norma/2760e4f16c8b4887976bc86b7076adf6/Lei_6475_2020.html	10
		Law No. 5.146	19/08/2013	It prohibits the sale and advertising of listed unhealthy products (including soft drinks, candies, and fried foods - among others) and mandates the daily offering of at least one variety of fruit. It provides for the training of faculty in nutritional education. It is valid for both public and private school systems.	Public/Private	https://goo.gl/7Y9ZiB	
		Decree No. 36.900	23/11/2015	It regulates Law 5.146/13, which prohibits the sale and advertising of listed unhealthy products (including soft drinks, candies, and fried foods - among others) and mandates the daily offering of at least one variety of fruit.	Public/Private	https://goo.gl/UFjNgr	

		Decree No. 37346	17/05/2016	Amends paragraph 2 of article 3 of Decree No. 36,900, of November 23, 2015, which regulates Law No. 5,146, of August 19, 2013, establishing guidelines for the promotion of adequate and healthy food in schools in the Federal District's education network, and provides other measures.	Public/Private	http://www.sinj.df.gov.br/sinj/Norma/14d039923cf848ac920cd005ea29954d/Decreto_37346_17_05_2016.html	
